# Induced abortion in Iran, Tehran University of Medical Sciences, the law and the diverging attitude of medical and health science students

**DOI:** 10.1371/journal.pone.0320302

**Published:** 2025-03-25

**Authors:** Mohammad Haddadi, Leyla Sahebi, Fatemeh Hedayati, Iqbal H. Shah, Mohammadamin Parsaei, Mamak Shariat, Mohadese Dashtkoohi, Sedigheh Hantoushzadeh

**Affiliations:** 1 Vali-E-Asr Reproductive Health Research Center, Family Health Research Institute, Tehran University Of Medical Sciences, Tehran, Iran; 2 Maternal, Fetal & Neonatal Research Center, Family Health Research Institute, Tehran University Of Medical Sciences, Tehran, Iran; 3 Department of Global Health and Population, Harvard T H Chan School of Public Health, Boston, Massachusetts, United States of America; 4 Breastfeeding Research Center, Family Health Research Institute, Tehran University of Medical Sciences, Tehran, Iran; Hamadan University of Medical Sciences, School of Public Health, IRAN, ISLAMIC REPUBLIC OF

## Abstract

**Background:**

The topic of induced abortion has been a subject of extensive debate among various moral and religious frameworks and continues to pose significant challenges within the domains of medical ethics and policy formulation. In the context of Iran, an Islamic republic, the approach to induced abortion has been notably influenced by historical, social, and political dynamics. Following the implementation of the ‘Rejuvenation of the Population and Protection of the Family Law (RPPF)’ in 2021, access to induced abortion and contraceptive measures has been markedly restricted as a response to concerns surrounding population decline.

**Objective:**

This study aims to assess the attitudes of Iranian medical and health science students toward induced abortion as future executives of health programs.

**Methods:**

This cross-sectional study administered a structured questionnaire for self-completion to medical and health science students of Tehran University of Medical Sciences, including medical students, obstetrics and gynecology residents, and nursing and midwifery students. The scoring of responses was between ‒12 and + 12, with the score ranging from 0 to positive, 12 showing an attitude negative to induced abortion, and the score below zero to ‒12 reflecting a positive attitude toward induced abortion.

**Results:**

A total of 237 participants were involved in the study, with 52% being female, and 60% originally from cities other than Tehran. The median (min, max) of the total score of the attitude toward induced abortion was ‒5.0 (12,10). The mean score varied by the age of the respondents, with ‒4.0 (‒12, 10) for those aged above 30 years compared to ‒5.0 (‒12,10) for those aged below 30 years (P-value = 0.043). The score was 0.0 (‒12,10) for married compared to ‒6.0 (‒12,10) among single participants (P-value < 0.001). The score for participants who agreed with RPPF was 0.0 (‒12,10), compared to a more positive attitude of ‒8.0 (‒12,9) for participants who disagreed (P-value < 0.001).

**Conclusion:**

Iranian medical and health science students support induced abortion before 16 weeks of gestation. The attitudes of medical students who are future providers of health care and implementers of RPPF and other health laws are, therefore, at variance with current laws and policies.

## Introduction

Abortion refers to the end of a pregnancy before the 20^th^ week of gestation, which can be spontaneous or induced [1]. Induced abortion refers to the medical or surgical termination of pregnancy, which can be done for various reasons and contexts [2]. The issue of induced abortion has long been disputed in various moral and religious schools and remains one of the most challenging issues in the field of medical ethics and policymaking. Currently, various countries have different approaches to abortion. Some countries, such as England, Netherlands, and Turkey, legally permit abortion up to the 24^th^ week of pregnancy on request. However, in many other countries, it is permitted only to save the life of the mother [3,4].

In the Islamic Republic of Iran, abortion law has seen some twists and turns. With the victory of the Islamic revolution in 1979, the legislators, emphasizing the religious teachings on the prohibition of induced abortion, enacted stricter laws on abortion access, and people who directly or indirectly participated in abortion faced criminal penalties. However, after 15 years, according to the Population Control Law of 1993, medical abortion in situations where the fetus has certain diseases that make the continuation of the fetus difficult or the mother has difficulties in keeping the fetus, or she has a disease that makes the pregnancy dangerous for her before the “soul enters the fetus” (16^th^ weeks of gestation due to Shiite scholars) was legally authorized by a specialist [5]. Contraceptive use in Iran rose high, and fertility declined to low levels. In the law of 2021, which was promulgated by the conservative government to increase the population, in addition to the restrictions in the field of prenatal screening, more restrictions were also placed on abortion, and the authorization of medical abortion was placed under the responsibility of a council consisting of a judge and a specialist doctor. In this way, the legislatures made the condition of medical abortion more stringent along with prenatal screening, and the lawbreakers have faced more severe criminal penalties [6].

Students in health domains, including medical, nursery, and midwifery, will gradually enter the country’s healthcare system in the next decade and will implement the health policies of the country. Therefore, ascertaining the attitudes of this group is necessary for the implementation of existing policies and for enacting more appropriate policies in the field of medical education. For example, A study in Jordan showed that medical students had a conservative approach to induced abortion [7], while a study in Bosnia and Herzegovina found that 81% of medical students supported abortion under certain conditions [8].

Induced abortion remains a highly debated and sensitive issue worldwide, with varying legal, ethical, and cultural perspectives. Understanding the attitudes of future healthcare providers, such as medical students, obstetrics and gynecology residents, and nursing and midwifery students, is crucial as they will play a pivotal role in reproductive health services. In this study, we aim to assess the attitudes of medical students, obstetrics and gynecology residents, and nursing and midwifery students toward induced abortion and compare it with the legal processes of the country and the current law of the population. We also ascertain the differentials in attitudes by background characteristics of respondents, such as age and marital status. By identifying these attitudes and their differentials based on background characteristics, the study aims to inform policymakers and educational institutions, ultimately contributing to more informed and empathetic healthcare practices.

## Method and materials

### Study design and population

This cross-sectional study was performed at the Tehran University of Medical Science from 1^st^ August 2023 to 1^st^ December 2023. The population was medical students, obstetrics and gynecology residents, and midwifery and nursing students studying at the university. International students and PhD students were excluded from this study. Medical students were divided into two groups (preclinic: years 1‒3 and clinic: years 4‒7).

The questionnaire developed by Sloan was used in this study [9]. In the first stage, permission was ascertained from Sloan to adapt and validate the questionnaire for this study. To validate, first, the translation validity was implemented and confirmed. For this, an expert translator translated the questionnaire questions into Farsi because Farsi is the national and official language in Iran. In the next step, the questions were translated into English again by another translator who was unaware of the original English version. Then, the original version of the questionnaire was compared with the new version to check and confirm the similarities between the two.

In the next step, face and content validity was determined. For this, the questions were evaluated by a group of eight clinical experts, psychiatrists, and epidemiologists. This way, the questionnaire was checked regarding its appearance (length, format, clarity, number, correct framing, etc.) based on the ABC of Face Validity for the Questionnaire [10]. 11 questions were asked to each expert, and they answered yes or no for each question in the questionnaire. After that, the agreement was calculated, and the mean of agreement between all experts was calculated. In the end, no significant changes occurred because the overall agreement for all questions was more than 90%.

The content validity ratio (CVR) technique was obtained by the Lawshe table [11] to check the quantitative validity of the content. Experts had three options for each item: (1) it is useful and necessary, (2) it is useful but not necessary, and (3) it is neither useful nor necessary. The eight experts voted, and the responses were measured by the following formula CVR, if any question was higher than 0.75, it remained in the questionnaire. Also, the Content Validity Index (CVI) was measured based on the Lawshe table. For the four questions with a CVR of 1, the item level CVI is 1. For the eight questions with a CVR of 0.75, the item level is 0.75. The average scale level of CVI of all items is 0.833, the universal scale level of CVI is 0.833., and the universal agreement is 33.33%. A CVI of more than 0.8 is appropriate in all scales [11].

The original questionnaire had 14 questions, with each question having six response options. We reduced the number of questions to 12 and included two main response options: 1) Agree, and 2) Disagree, for clarity and to avoid confusion. An additional “No idea” option was provided for participants who were unsure about the question.

All these finalized 12 questions had a CVR of more than 0.75. It is shown in the supplementary S1 Table. Finally, these questions were pre-tested with a group of 15 students. Its internal reliability was evaluated with a split-half, and a question was removed if it has a high dispersion of below 0.7. The internal reliability of 12 questions was 0.776. Also, face and content validity was evaluated among these 15 students in the second step based on the ABC of Face Validity for the Questionnaire [10]. During this phase, similar to the initial evaluation among the expert group, each student was presented with 11 questions, answering either yes or no. Subsequently, the level of agreement was calculated, and the mean agreement across all experts was determined. Ultimately, no significant changes were observed, as the overall agreement for all questions exceeded 90%.

After finalizing the Farsi version, it was self-completed by the respondents. Convenience sampling was considered in this study through classrooms in the university and hospitals. They completed the questionnaire in the classrooms after declaring informed consent. Before that, an expert medical doctor who was familiar with the questionnaire details and the validation process provided an explanation of the questionnaire to the students. Each questionnaire had an individual number, and students did not need to write their name and identity document on the forms.

The sample size was determined based on a pilot study conducted with 15 medical students and residents in the obstetrics and gynecology (OB/GYN) morning shift, using convenience sampling. The pilot study aimed to estimate the effect size for the total attitude score in relation to the presence of 9 confounding variables.

The effect size for the linear regression analysis was calculated based on the pilot study, which yielded an effect size of 0.1. The sample size calculation was performed using the G * Power software, assuming a linear regression test with 9 confounding variables. A design effect of 0.2 was incorporated to adjust for potential clustering or stratification. Based on these parameters, the required sample size was estimated to be 230 participants to achieve adequate statistical power. The total number of eligible students was 3700. The questionnaire was explained to 400 students in the classrooms. The larger sample was intended to account for potential dropouts, non-responses, and incomplete data, which are common in studies involving surveys. Two hundred fifty students were accepted to complete the questionnaire, and 237 questionnaires were complete and eligible to be involved in the study.

### Outcomes

The primary outcome was to assess the attitudes of students of nursing, medicine, and midwifery toward induced abortion before 16 weeks of gestation. The secondary outcomes investigated the association between the background characteristics of respondents and attitudes.

### Data collection and measurements

The final questionnaire contained 12 questions. Each question provides three response options: agree, disagree, and no idea. Agreeing with questions numbered 1, 3, 4, 5, 7, 10, and 12 are against induced abortion (pro-life). While agreeing with other questions means agreeing with induced abortion (pro-choice). If participants agreed with pro-life questions, it was scored + 1, if they had no idea, it scored 0, and if they disagreed, it was scored ‒1. Conversely, if participants agreed with pro-choice questions, it was scored ‒1, if they had no idea, it was scored 0, and if they disagreed, it scored + 1. Therefore, the total scores for each participant are between ‒12 and + 12, where + 12 means strongly pro-life and against induced abortion, while ‒12 means strongly pro-choice and supporting induced abortion. The higher the score from 0 to positive 12, the greater the attitude to oppose induced abortion, and the lower the score to minus 12, the greater the attitude to agree with induced abortion. Also, background characteristics, including age, sex, previous and present number of family members, level of education, current residence, residence before entering the university, level of familiarity with the new abortion law, religion (Shia or Sunni), marriage status, and children, was documented.

The environment for data collection consisted of a classroom setting, which was designed to ensure comfort and minimize distractionls during the survey process. The classrooms were private to allow participants to respond without interruptions.To ensure confidentiality and encourage candid responses, participants were assured that their answers would remain anonymous and that no identifying information would be collected. Additionally, they were informed that their participation was voluntary and that they could withdraw at any time without consequence. Measures were in place to prevent peer influence, as participants were given time to complete the survey individually and in a private setting.

### Statical analysis

Descriptive statistics was used to describe demographic characteristics, awareness, and attitudes towards abortion. The Kolmogorov-Smirnov test was used to check for normal distribution of data. All quantitative variables in this study had non-normal distribution. Therefore, we demonstrated quantitative data in median (minimum-maximum), and we used the Mann-Whitney test and Kruskal Wallis test to investigate the relationship between characteristics and final scores. These non-parametric tests were chosen because the data did not meet the assumption of normality, as assessed by visual inspection of histograms and formal statistical tests (e.g., Shapiro-Wilk test). Given that both tests are robust to violations of normality and are suitable for comparing ordinal or non-normally distributed continuous data, they were deemed appropriate for the analysis of our data. The analysis was performed using the statistical package “SPSS” version 25. The significance level for all statistical tests was set at P < 0.05.

### Ethical considerations

The Ethical Committee of the Tehran University of Medical Science approved the study (IR.TUMS.IKHC.REC.1402.084), and informed written consent was obtained from all participants. The study was conducted according to the declaration of Helsinki [12].

## Results

The participation rate in this study was 59.25%, with 237 out of 400 students who were approached completing and submitting eligible questionnaires with median age of 25 years (min:18, max:45 years). Among the participants, 168 (70.9%) were medical students, 63 (26.6%) were preclinical, and 105 (44.3%) were clinical. In addition, 34 (14.3%) were obstetrics and gynecology residents, 16 (6.8%) were nursery students, and 19 (8.0%) were midwifery students. Among participants, 31 (13.1%) participants were above 30 years old, 37 (15.6%) were married, and 222 (93.7%) declared their religion as Shia. Moreover, 103 (43.5%) lived in the dormitory, and 82 (34.6%) lived with their family in Tehran. When the participants were asked about the Rejuvenation of the Population and Protection of Family Law, 106 (44.7%) knew the law, 52 (49.1%) of them were against the law, while 31 (29.2.9%) participants were neutral. The background characteristics are shown in Table 1. The scattering of answers to each question in detail is shown in Table 2.

**Table 1:** The Background Characteristic of Students in Tehran University of Medical Sciences, 2023.

Variables		N (%)
Age (>30)		31 (13.1)
Sex (female)		125(52.7)
Family number (before intending University (<=3)		48 (20.3)
Marital status (married)		37(15.6)
Child (Yes)		24(10.1)
Current residency	Dormitory	103(43.5)
	Family	82(34.6)
	Partner	29(12.2)
	Alone	23(9.7)
Residency before intending University	Tehran	94(39.7)
	Other cities	141(59.5)
	Rural	1(0.4)
Religion	Shia	222(93.7)
	Sunni	5(2.1)
	Others	10(4.2)
Knowledge about RPPF^*^ (Yes)		106(44.7)
Idea about RPPF^*^^,^^**^	Agree	23(21.7)
	Disagree	52(49.1)
	No idea	31(29.1)
Field of Study	Medical students (preclinical)	63(26.2)
	Medical students (clinical)	105(44.3)
	Obstetrics and gynecology residents	34(14.3)
	Midwifery students	19(9.1)
	Nursing Students	16(6.7)

All data are demonstrated in number (%), Total number of respondents is 237.

* : RPPF: The Rejuvenation of the Population and Protection of the Family Law.

** : the total number is 106 participants who knew the law.

**Table 2:** The Scattering of Answers to Each Question of The Questionnaire among Students in Tehran University of Medical Sciences, 2023.

Question	Answers		
	agree	disagree	No idea
**1. Abortion should be considered a criminal act by the judicial system.**	38(16.0)	172(72.6)	27(11.7)
**2. Abortion is a good solution for unwanted pregnancy.**	120(50.6)	84(35.4)	33(13.9)
**3. The mother should feel the duty to give birth to the child she conceives**	67(28.3)	119(50.2)	51(25.5)
**4. Abortion is wrong. It doesn’t matter what the context is.**	17(7.2)	199(84.0)	20(8.9)
**5. Every fetus has the right to be born.**	44(18.6)	148(62.4)	45(19.0)
**6. A pregnant woman who does not want her child should be encouraged to have an abortion.**	48(20.3)	131(55.3)	58(24.5)
**7. Abortion should be considered as killing a person.**	36(15.2)	163(68.8)	38(16.0)
**8. Women who have chosen to have an abortion should not be looked down upon.**	171(73.0)	33(13.9)	31(13.1)
**9. Abortion should be an accessible option for teenagers who become pregnant outside of marriage.**	151(63.7)	48(20.3)	38(16.0)
**10. People should not decide on the life or death of the fetus.**	49(20.7)	136(57.4)	52(21.9)
**11. An unwanted child should not be born.**	67(28.3)	104(43.9)	66(27.8)
**12. Fetus should be considered as a person (human) after conception.**	62(26.2)	124(52.3)	51(25.5)

All data are demonstrated in number (%t), Total number of respondent is 237 participants. All question were asked based on induced abortion before 16 weeks of gestation.. Agreeing with questions numbered 1, 3, 4, 5, 7, 10, and 12 are against induced abortion (Pro-life), While agreeing with other question means ageing with induced abortion (Pro-choice).

The median (min, max) of the total score of the attitude toward induced abortion among participants was ‒5.0 (‒12,10). The score associated with participants aged above 30 years was ‒4.0 (‒12,10), while it was ‒5.0 (‒12,10) for those below 30 years (P-value = 0.043). The score for females and males was, respectively, ‒4.0 (‒12,10) and ‒6.0 (‒12,10) (P-value = 0.062). The total score among married participants was 0.0 (‒12,10), compared to ‒6.0 (‒12,10) among single participants (P-value < 0.001). The total score for participants without any children was ‒5.0 (‒12,10) and 0.0 (‒4,8) among participants with one child as compared to the score of ‒4.5 (‒12,10) for participants with more than two children (P-value = 0.047). Moreover, the scores of participants living in the dormitory, with family, with a partner, and alone were ‒6.0 (‒12,10), ‒5.0 (‒12,9), ‒1.0 (‒9,10), ‒6.0 (‒12,10) respectively (P-value = 0.004). Furthermore, the total score among participants agreeing with The Rejuvenation of the Population and Protection of the Family Law was 0.0 (‒12,10), whereas it was ‒7.0 (‒12,9) for participants disagreeing with the law and ‒5.0 (‒12,10) for those with no opinion about the law (P-value = 0.001). The score of attitude toward abortion is shown in Table 3.

**Table 3:** The Attitude Toward Abortion Based on Total Score of Students in Tehran University of Medical Sciences, 2023.

variables		Scores	P-vale
Total		‒5.0(‒12,10)
Age^*^	>30 years	‒4.0(‒12,10)	0.043
	< 30 years	‒5.0(‒12,10)	
Sex^*^	Female	‒4.0(‒12,10)	0.062
	Male	‒6.0(‒12,10)	
Family number (before intending university)^*^	<=3	‒4.0(‒12,10)	0.100
	>3	‒6.0(‒12,10)	
Marital statues	Married	0.0(‒12,10)	<0.00
	Single	‒6.0(‒12,10)	
Number of children^**^	0	‒5.0(‒12,10)	0.047
	1	0.0(‒4,8)	
	>=2	‒4.5(‒12,10)	
Current residency^**^	Dormitory	‒6.0(‒12,10)	0.004
	Family	‒5.0(‒12,9)	
	Partner	‒1.0(‒9,10)	
	alone	‒6.0(‒12,10)	
Residency before intending university^*^	Tehran	‒4.0(‒12,9)	0.382
	Other cities	‒6.0(‒12,10)	
Religion^**^	Shia	‒5.0(‒12,10)	0.324
	Sunni	‒8.0(‒10,‒2)	
	Others	‒5.5(‒12,‒1)	
Knowledge of RPPF^*1^	No	‒5.0(‒12,10)	0.968
	Yes	‒5.0(‒12,10)	
Idea about RPPF^**1^	Agree	0.0(‒12,10)	0.001
	Disagree	‒7.0(‒12,9)	
	No idea	‒5.0(‒12,10)	
Field of Study^**^	Medical students (preclinical)	‒5.0(‒12,10)	0.127
	Medical students (clinical)	‒6.0(‒12,10)	
	Obstetrics and gynecology residents	‒4.0(‒12,10)	
	Midwifery and Nursing students	‒4.0(‒12,9)	

All data is shown in median (minimum, maximum),. The higher the score goes from 0 to positive 12, the greater the attitude is to oppose induced abortion, and the lower the score goes to minus 12, the greater the attitude is to agree with induced abortion.

* : Mann-Whitney test is used, ^**^: Kruskal Wallis test is used, P-value < 0.05 is significant

^1^: RPPF: The Rejuvenation of the Population and Protection of the Family Law

## Discussion

The results of this survey show that the general attitude toward induced abortion before 16 weeks of gestation among medical students, obstetrics and gynecology residents, midwifery, and nursing students is positive and pro-choice. However, married people, those who live with their spouse, and those who agree with the Rejuvenation of the Population Law are less supportive of induced abortion. These results indicate that Iranian medical students who are supportive of abortion and will be responsible for health care in the future might face challenges regarding the implementation of the Rejuvenation of the Population Law, which restricts the provision of abortion and contraception to the general population.

While the study sheds light on the current state of attitudes toward abortion among healthcare students in Iran, it is important to consider the underlying causes and factors that may influence these perspectives. The cultural context in Iran, which places a strong emphasis on family values and childbearing, may also contribute to the more conservative stance on abortion observed among certain demographic groups in the study, such as married students and those with children. The influence of medical education, clinical experience, and exposure to ethical discussions in the field of reproductive health may also shape students’ opinions, particularly as they prepare to become healthcare providers responsible for making decisions about reproductive healthcare [13].

The main reasons for restricting abortion historically were to protect women from the dangers of illegal abortion and the unskilled abortionists who can harm them, to enforce moral standards and discourage abortion as a sin, and to safeguard fetal life in some or all cases [5,14,15]. United Nations reported that in 2017, almost every country in the world permitted abortion to protect a woman’s life; however, those involved in an unlawful abortion can face criminal charges according to explicit provisions in most countries [14]. Iran had a fluctuating history regarding abortion. After the establishment of the modern legal system, the act of abortion was criminalized in 1926 and had heavy punishments except for saving the mother’s life. The first decriminalization of the act of abortion was done in 1969, which justified abortion not only in the time of preserving a mother’s life but also in cases of preserving the mother’s health. Later, in 1976, the law permitted unrestrictive abortion before 12 weeks of gestation based on the parents’ decision and by providing sufficient reasons. Also, therapeutic abortion was permitted without any restriction based on the parent’s request and physicians’ judgments, and only the mother’s decision was required. After the 1979 revolution, abortion was again criminalized in 1982 due to Shia jurisprudence, with the punishment of paying blood money [5]. Islamic scholars permit induced abortion just before the point of ensoulment (120 days); however, there are controversies and disagreements about the permitted situations before 120 days of gestation [16]. Until 1998, only abortion for saving a mother’s life was legally permitted. After 2005, the Therapeutic Abortion Act and induced abortion were permitted only in case of problematic fetuses and major anomalies [5]. In 2021, after The Rejuvenation of the Population and Protection of the Family (RPPF) law, different types of contraceptives were previously restricted, with access limited to certain individuals based on health conditions and previous children, such as vasectomy and obtaining an abortion became more difficult than before. A team of Muslim doctors, a forensic medicine expert, and a judge must approve a pregnant woman’s abortion request, and judges have the final authority. They can allow the abortion if the fetus is under four months old, the mother or the fetus has a life-threatening condition or a severe disability, and there is no other reason to permit abortion before the point of ensoulment [6].

Attitudes to induced abortion are different in countries due to many factors like socio-economic situations, religion, political ideology, and the reasons under which abortion is legally permitted. While most people are in favor of therapeutic abortion, there is less support for elective abortion, where the woman chooses to end the pregnancy. A study in the United States and New Zealand found that while support for elective abortion has gradually increased over the past few decades, a considerable minority of the public remains opposed to it [17]. This gradual shift is in contrast to the more static views on abortion in Iran, where restrictive policies such as the Rejuvenation of the Population and Protection of the Family (RPPF) Law continue to shape public opinion. Despite this, our study found that medical students in Iran, who will play a pivotal role in future healthcare policies, are more supportive of abortion than the general public, possibly indicating a generational divide. This mirrors trends observed in Western countries, where younger generations tend to favor reproductive rights more than older generations.

It is difficult to access the exact data on abortion in Iran because of the challenge of reaching all the different groups of people and the delicate nature of the issue. The study by Mahdavi et al. indicated that no abortion permission was granted for non-medical cases, which likely reflects the restrictive nature of abortion laws in Iran. [18]. However, this study did not assess the attitudes of medical students or healthcare providers toward such laws, a gap our study aims to address. In contrast to our study’s focus on medical students, the study by Hosseini et al. looked at a broader female population, showing that 3.8% of women aged 15–49 had an induced abortion. The difference may arise from the broader societal factors affecting abortion, such as cultural norms and policies [19]. However, a survey by Ranji has shown that 17% of women had experienced at least one illegal induced abortion, and having an abortion was mainly due to the desire to prevent or defer childbearing and the economic problems of the family. Non-medical providers unsafely do a considerable amount of these abortions [20]. Abortion complications are much lower in safe abortions than in unsafe abortions [21]. The RPPF law, which limits access to abortion, may inadvertently lead to an increase in illegal abortions. Policymakers must consider these public health risks when shaping future legislation, ensuring that the legal framework protects women’s health and reduces the potential harms of unsafe abortion practices.

Studies around the world have shown different attitudes toward induced abortion in medical students. A study conducted among Jordanian medical and health sciences students found that abortion was largely viewed negatively, except in cases where the mother’s life was at risk or the pregnancy was a result of rape. This is consistent with the more conservative stance observed in many Middle Eastern countries, where cultural and religious factors heavily influence attitudes toward abortion [7]. Our findings, however, suggest a more pro-choice attitude among Iranian medical students, which may reflect a generational shift or differing regional attitudes, though factors such as marital status and legal constraints still play a significant role. These differences highlight the complex intersection between individual beliefs, societal norms, and national policies on abortion.. In Bosnia and Herzegovina, 87.8% of medical students who participated in the study agreed that abortion is “murder,” and only a few of them would perform an abortion on request. However, medical conditions were acceptable reasons for many students to perform an abortion [8]. In South Africa, a study revealed that while most medical students held positive attitudes toward medical abortion, only a small percentage (12.5%) would perform or refer for abortion on request. [22]. This finding is consistent with our own results, where medical students expressed support for abortion in certain circumstances, such as to preserve the mother’s health or in the case of fetal anomalies. However, like South African students, Iranian medical students showed reluctance to support elective abortion on demand. This may be partly due to the religious and legal framework in Iran, where abortion is heavily restricted by law, influencing the views of even future healthcare providers.

However, few medical students at the University of Washington thought that 1st-trimester abortion should not be accessible, and the overall attitudes toward 1st-trimester abortion were more positive than 2nd-trimester abortion [23]. Furthermore, 62% of medical students in the United Kingdom had pro-choice attitudes [24], and it is believed that medical education should train students to be skilled practitioners, with clinical placements and non-stigmatizing abortion care as critical components. They proposed that teaching should be for everyone, even those who object to abortion on conscience grounds [25].

In this study, marriage status and living with a spouse were factors associated significantly with more pro-choice abortion attitudes. Ranj has found that education level, religion, family income, ethnicity, number of children, and age at marriage are related to having an induced abortion [20]. The study by Rosenblatt et al. found that older students were more supportive of abortion, which aligns with our finding that participants over 30 years old were more likely to oppose abortion [23]; also, Osborne et al. demonstrated that abortion support for women and men is more among men and is influenced by both their religiosity and their intimate partners’ religiosity [17]. Meanwhile, there was no statistical evidence of the effect of medical education length and sex on abortion attitudes among Bosnia and Herzegovina students [8]. In our study, men were more supportive of induced abortion, while it was not statistically significant. Moreover, in our study, people aged more than 30 years are more against induced abortion compared to younger. Most of the population in our study were Shia, and data could not demonstrate the comparison between religious declaration and abortion attitudes.

This study was a rare investigation of the induced abortion attitude in Iran. The respondents were young people who were responsible for health decisions. The study shows how the laws and policies are at variance with the perspectives and attitudes of medical students who are future healthcare providers in Iran. The study has some limitations. First, the lack of students with other religions besides Shia did not allow us to compare students’ attitudes with varying religions. Future research that includes participants from a wider variety of religious backgrounds will be essential to provide a more comprehensive understanding of how religious diversity influences attitudes toward abortion in Iran.

Secondly, some students refused to participate due to sociopolitical situations. We emphasized confidentiality and ethical consideration of privacy and informed consent, which helped to improve the response rate. The findings are limited to students of the Tehran University of Medical Science and may not be generalizable to students of other medical science universities in Iran, and the results might be influenced by the specific practices, culture, and environment of that particular institution, We did not investigate the different types and causes of induced abortion; which is another limitation in this study. Furthermore, one of the limitations of this study is the use of convenience sampling, which may introduce selection bias. This sampling method may not fully represent the broader population, potentially limiting the generalizability of the findings, and the findings may not be directly applicable to other populations or settings. Future studies employing random sampling methods may help mitigate selection bias and improve the external validity of the results. Finally,

## Conclusion

In conclusion, this study has highlighted that medical and health science students in Iran generally exhibit positive attitudes toward induced abortion. However, factors such as marital status, having children, and cohabitation with a partner seem to contribute to a more conservative stance on the issue. These findings are significant in the context of Iran, where abortion laws are heavily influenced by Islamic principles and are limited to specific medical circumstances. The disconnect between students’ attitudes and the national policies outlined in the RPPF law poses a substantial challenge for the future implementation of abortion-related policies. Specifically, the younger generation of future healthcare providers, who may be responsible for enforcing these laws, shows a trend toward more progressive views on reproductive rights. Aligning public health initiatives with the evolving views of medical students and professionals is essential for developing more effective and socially acceptable healthcare policies related to reproductive rights in Iran. These findings suggest that incorporating broader ethical discussions, clinical experiences, and education on reproductive health into medical training could help reconcile these differing views and improve the overall healthcare framework.

## Supporting information

S1 Table The CVR evaluation of the Questionnaire. (DOCX)

## References

[1] Abortion. Definition, procedure, laws, & facts. Britannica. 2023. https://www.britannica.com/science/abortion-pregnancy

[2] Kapp N, Lohr PA. Modern methods to induce abortion: Safety, efficacy and choice. Best Pract Res Clin Obstet Gynaecol. 2020;63:37–44. doi: 10.1016/j.bpobgyn.2019.11.008 32029379

[3] Shapiro GK. Abortion law in Muslim-majority countries: an overview of the Islamic discourse with policy implications. Health Policy Plan. 2014;29(4):483–94. doi: 10.1093/heapol/czt040 23749735

[4] Singh K, Ratnam SS. The influence of abortion legislation on maternal mortality. Int J Gynaecol Obst. 1998;63(Suppl 1):S123–129. 10075222 10.1016/s0020-7292(98)00194-5

[5] Abbasi M, Shamsi Gooshki E, Allahbedashti N. Abortion in Iranian legal system: a review. Iran J Allergy Asthma Immunol. 2014;13(1):71–84. 24338232

[6] Aramesh K. Population, abortion, contraception, and the relation between biopolitics, bioethics, and biolaw in Iran. Develop World Bioethics. 2023. 10.1111/dewb.12386 36649588

[7] Saadeh R, Alfaqih M, Odat A, Allouh M. Attitudes of medical and health sciences students towards abortion in Jordan. Biomed Res Int. 2021;2021:6624181. doi: 10.1155/2021/6624181 33997028 PMC8096537

[8] Trninić Z, Bender M, Šutalo N, Kozomara D, Lasić V, Bevanda D, et al. Attitudes of students of medicine, university of mostar according to induced abortion. Psychiatr Danub. 2017 Dec;29;Suppl 4(Suppl 4):866–71. 29278638

[9] Sloan LA. Abortion attitude scale. Health Educ. 1983;14(3):41–2. 6443921

[10] 25.pdf [Internet]. 2024 [cited 2024 Sep 15]. Available from: https://globalresearchonline.net/journalcontents/v65-1/25.pdf

[11] LAWSHE CH. A quantitative approach to content validity1. Person Psychol. 1975;28(4):563–75. doi: 10.1111/j.1744-6570.1975.tb01393.x

[12] Goodyear M, Krleza-Jeric K, Lemmens T. The declaration of Helsinki. BMJ. 2007;335(7621):624–5. 17901471 10.1136/bmj.39339.610000.BE PMC1995496

[13] Liu C-I, Tang K-P, Wang Y-C, Chiu C-H. Impacts of early clinical exposure on undergraduate student professionalism-a qualitative study. BMC Med Educ. 2022;22(1):435. doi: 10.1186/s12909-022-03505-5 35668444 PMC9172165

[14] World Population Policies. Population Division [Internet]. [cited 2024 Feb 24]. Available from: https://www.un.org/development/desa/pd/data/world-population-policies

[15] Berer M. Abortion law and policy around the world: in search of decriminalization. Health Hum Rights. 2017;19(1):13–27. 28630538 PMC5473035

[16] K A. ABORTION. An islamic ethical view. 2007 Jan 1;6(5):29–33 [cited 2024 Feb 24]. Available from: https://www.sid.ir/paper/291152/fa

[17] Osborne D, Huang Y, Overall NC, Sutton RM, Petterson A, Douglas KM, et al. Abortion attitudes: an overview of demographic and ideological differences. Political Psychol. 2022;43(S1):29–76. doi: 10.1111/pops.12803

[18] Mahdavi SA, Jafari A, Azimi K, Dehghanizadeh N, Barzegar A. Therapeutic abortion in Iran: an epidemiologic study of legal abortion in 2 years. BMC Res Notes. 2020 May 27;13(1):261 [cited 2024 Feb 24]. Available from: doi: 10.1186/s13104-020-05098-y 32460874 PMC7254741

[19] Hosseini H, Erfani A, Nojomi M. Factors associated with incidence of induced abortion in hamedan, Iran. Arch Iran Med. 2017;20(5):282–7. 28510463

[20] Ranji A. Induced abortion in Iran: prevalence, reasons, and consequences. J Midwifery Womens Health. 2012;57(5):482–8. doi: 10.1111/j.1542-2011.2012.00159.x 22954079

[21] Bridwell RE, Long B, Montrief T, Gottlieb M. Post-abortion complications: a narrative review for emergency clinicians. West J Emerg Med. 2022;23(6):919–25. doi: 10.5811/westjem.2022.8.57929 36409940 PMC9683756

[22] Buga GAB. Attitudes of medical students to induced abortion. East Afr Med J. 2002;79(5):259–62. doi: 10.4314/eamj.v79i5.8865 12638811

[23] Rosenblatt RA, Robinson KB, Larson EH, Dobie SA. Medical students’ attitudes toward abortion and other reproductive health services. Fam Med. 1999;31(3):195–9. 10086256

[24] Gleeson R, Forde E, Bates E, Powell S, Eadon-Jones E, Draper H. Medical students’ attitudes towards abortion: a UK study. J Med Ethics. 2008;34(11):783–7. doi: 10.1136/jme.2007.023416 18974410

[25] Horan C, Zadeh PG, Rennison C, Hoggart L, Kavanagh J. A qualitative analysis of medical students’ attitudes to abortion education in UK medical schools. BMJ Sex Reprod Health. 2022;48(3):205–9. doi: 10.1136/bmjsrh-2021-201385 35102002

