# Supplementary material for: Induced abortion in Iran, Tehran University of Medical Sciences, the law and the diverging attitude of medical and health science students
Source: PLoS One. 2025 Mar 25;20(3):e0320302. doi: 10.1371/journal.pone.0320302 (PMC11936268; doi:10.1371/journal.pone.0320302)
Supplement: S1 Table — (DOCX) [file pone.0320302.s001.docx]

Supplementary Table 1. The CVR evaluation of the Questionnaire

| The CVR | | | |  |
| --- | --- | --- | --- | --- |
| Question | Answers | | |  |
|  | it is useful and necessary | it is useful but not necessary | it is neither useful nor necessary | CRV |
| 1. Abortion should be considered a criminal act by the judicial system. | 8 | 0 | 0 | 1 |
| 2. Abortion is a good solution for unwanted pregnancy. | 8 | 0 | 0 | 1 |
| 3. The mother should feel the duty to give birth to the child she conceives | 8 | 0 | 0 | 1 |
| 4. Abortion is wrong. It doesn't matter what the context is. | 8 | 0 | 0 | 1 |
| 5. Every fetus has the right to be born. | 7 | 1 | 0 | 0.75 |
| 6. A pregnant woman who does not want her child should be encouraged to have an abortion. | 7 | 1 | 0 | 0.75 |
| 7. Abortion should be considered as killing a person. | 7 | 1 | 0 | 0.75 |
| 8. Women who have chosen to have an abortion should not be looked down upon. | 7 | 1 | 0 | 0.75 |
| 9. Abortion should be an accessible option for teenagers who become pregnant outside of marriage. | 7 | 1 | 0 | 0.75 |
| 10. People should not decide on the life or death of the fetus. | 7 | 1 | 0 | 0.75 |
| 11. An unwanted child should not be born. | 7 | 0 | 0 | 0.75 |
| 12. Fetus should be considered as a person (human) after conception. | 7 | 1 | 0 | 0.75 |
| Total participant 8, due to CVR formula, more than 0.79 is acceptable | | | |  |
